# The Immediate Impact of Infarct Size on the Systemic Inflammatory Response: IL-6 as Central Mediator Identified through Biomarker and Proteomic Profiling

**DOI:** 10.1007/s12265-026-10801-8

**Published:** 2026-06-29

**Authors:** Jonathan Los, Frans B. Mensink, Özlem Bulut, Ilse H. Hol, Aysun Cetinyurek-Yavuz, Niels P. Riksen, Saloua El Messaoudi, Jan H. Cornel, Robert-Jan M. van Geuns

**Affiliations:** 1 Department of Cardiology, Radboud University Medical Center, Geert Grooteplein Zuid 10, 6525 GA Nijmegen, the Netherlands; 2 Department of Internal Medicine, Radboud University Medical Center, Nijmegen, the Netherlands; 3 Department of IQ Health, Radboud University Medical Center, Nijmegen, the Netherlands; 4 Department of Cardiology, Northwest Clinics, Alkmaar, the Netherlands; 5 Dutch Network for Cardiovascular Research (WCN), Utrecht, the Netherlands

**Keywords:** Myocardial infarction, Inflammation, C-reactive protein, Interleukin-6, Cytokines

## Abstract

Myocardial infarction (MI) induces an inflammatory response that may be amenable to targeted therapy. This cohort study evaluated the systemic inflammatory burden across different infarct sizes using serial measurements of C-reactive protein (CRP), interleukin-6 (IL-6), and targeted inflammatory proteomics (Olink’s Target 96 Inflammation panel) during hospitalization and up to 12 weeks post-MI. Baseline IL-6 concentrations were progressively higher with increasing infarct size, with differential temporal trajectories observed across infarct size strata (*P* for interaction < 0.001), a finding confirmed by proteomic analysis (*P*_*adj*_ for interaction = 0.009). Patients with small infarct size showed no significant changes in CRP or IL-6 concentrations, yet demonstrated downregulation of urokinase (uPA) and upregulation of angiogenesis-related proteins (tumor necrosis factor-related weak inducer of apoptosis [TWEAK] and hepatocyte growth factor [HGF]), similar to those observed in patients with moderate or large infarct sizes, emphasizing the biological significance of these processes. Overall, greater myocardial injury was associated with higher inflammatory burden, highlighting IL-6 as a promising therapeutic target for acute anti-inflammatory treatment after large MI.

**Supplementary Information:**

The online version contains supplementary material available at 10.1007/s12265-026-10801-8.

## Highlights

Among patients with myocardial infarction (MI), greater myocardial necrosis is associated with a higher inflammatory burden, particularly involving interleukin-6 (IL-6).

Given the pivotal role of IL-6 in atherosclerosis, our findings support IL-6 as potential therapeutic target for anti-inflammatory therapy and suggest that patients with larger myocardial injury may derive greater benefit.

During the acute phase, reduced expression of urokinase and upregulation of angiogenesis-related proteins was observed across all infarct sizes, emphasizing the biological significance of these processes even in patients with small MI.

**Graphical Abstract:**

Abbreviations: HGF, hepatocyte growth factor; IL-6, interleukin 6; MI, myocardial infarction; TWEAK, tumor necrosis factor-related weak inducer of apoptosis

**Supplementary Information:**

The online version contains supplementary material available at 10.1007/s12265-026-10801-8.

## Graphical abstract

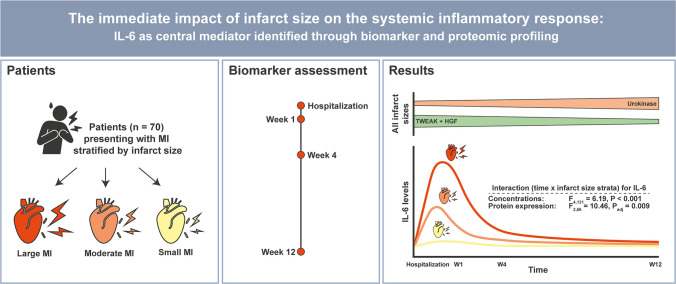

## Introduction

Myocardial necrosis induces an inflammatory response, characterized by cytokine signaling and leukocyte recruitment to orchestrate tissue healing [1, 2]. However, an excessive inflammatory cascade seems to have deleterious effects [1, 2]. Following an acute coronary syndrome (ACS), elevated concentrations of circulating inflammatory biomarkers are associated with adverse clinical outcome [3–6]. In particular the interleukin-1 (IL-1)—IL-6 pathway is increasingly recognized for its pivotal role in driving inflammation [1, 7]. Building upon these findings, the ASSAIL-MI trial demonstrated that tocilizumab, an IL-6 antagonist, ameliorates myocardial damage on cardiac magnetic resonance imaging when administered within a few hours of symptom onset [8]. Nonetheless, anti-inflammatory interventions may also interfere with host defense and tissue healing. The acute overshoot of cytokine levels following myocardial infarction (MI) might be an optimal time window to attenuate the inflammatory burden. A patient-tailored approach, by selecting patients with an excessive inflammatory response, seems most appropriate and advocated in recent perspectives [1]. Patients experiencing large MI may exhibit more pronounced elevations in cytokine levels and might benefit most from prompt anti-inflammatory therapy, whereas patients with minimal myocardial injury might show no systemic inflammatory response. In this exploratory study, we performed a comprehensive analysis on the inflammatory biomarker response over the first three months following MI, comparing patients with varying infarct sizes. Our aim was to characterize the inflammatory burden in detail and to describe whether patients with large myocardial injury demonstrate distinct inflammatory signatures.

## Methods

### Study Design

This study includes 70 consecutive patients (56 [80.0%] males and 14 [20.0%] females) admitted for ST-elevation myocardial infarction (STEMI) or non-ST-elevation myocardial infarction (NSTEMI) to the Radboud university medical center, Nijmegen, the Netherlands, who participated in the FITTER trial. The FITTER trial (NCT04141579) was designed to assess the immediate effect of very-high intensive lipid-lowering therapy on intracoronary non-culprit plaque features [9–11]. In the FITTER trial, patients presenting with ACS and multivessel disease were randomized to a proprotein convertase subtilisin/kexin 9 (PCSK9) inhibitor or placebo for 12 weeks in addition to high-intensity statin therapy. Aside from the primary outcomes, the trial’s brief follow-up period with frequent study site visits facilitated assessment of the systemic inflammatory burden in these patients following MI. Out of all patients included in the FITTER trial, only patients included at the Radboud university medical center underwent serial blood sampling for biomarker analysis. All patients in the present study presented with a type 1 MI. Only patients with complete inflammatory biomarker assessment were included in the analysis. Ethical approval was given by the Dutch Ethical Review Board (METC Oost Nederland) and the trial was conducted in full accordance with the principles of the “Declaration of Helsinki.” All participants provided written informed consent. Details about the study protocol, inclusion and exclusion criteria, and follow-up were reported previously [9].

### Blood Sampling and Assessment of Circulating Inflammatory Markers

Serial creatine kinase (CK) levels were obtained at local hospitals using site-specific analytical methods at 6-h intervals following clinical presentation until peak values were achieved. Peak CK levels were used as a surrogate measure of infarct size, as they have been demonstrated to correlate strongly with final infarct size on cardiac imaging [12–14]. Inflammatory biomarker assessment included measurements of circulating C-reactive protein (CRP) and IL-6, as well as comprehensive biomarker evaluation using Olink’s Proximity Extension Assay technology (Olink Proteomics, Uppsala, Sweden). Blood sampling was performed at multiple timepoints. Blood was obtained in BD Vacutainer® K2-EDTA tubes (BD Biosciences, San Jose, USA) at index hospitalization (baseline), week 4, and week 12. During the index hospitalization, the first blood sample was drawn after successful percutaneous coronary intervention of the culprit lesion. Plasma was acquired from the EDTA tubes after centrifugation (2744 g for 10 min at room temperature) and stored at −150 ◦C until the end of the study for simultaneous biomarker analysis. In plasma samples, CRP was quantified using DuoSet ELISA kits (Bio-Techne/R&D Systems, Minneapolis, USA), and IL-6 was measured using the Quantikine ELISA kits (Bio-Techne/R&D Systems, Minneapolis, USA), according to the manufacturer’s instructions.

Alongside the measurement of CRP and IL-6 concentrations, a more extensive targeted proteomics assessment was performed in plasma samples obtained at baseline and 12-week follow-up using Olink’s targeted proteomics technique, employing the inflammation panel according to the manufacturer’s protocol. The results of the measured Olink Target proteins are expressed as Normalized Protein eXpression (NPX) values, which represent relative protein abundance on a log2 scale. As the targeted proteomics panel was conducted in two separate runs, an additional normalization procedure using eight bridging samples was implemented to ensure that the data were directly comparable. Specifically, the median protein abundance for each protein was calculated across the bridging samples and used to correct one of the two data runs. Olink target proteins of which more than 80% of the sample results were below the adequate detection limit in either run, were removed from the analysis.

During the 12-week follow-up period, patients were intensively monitored for adverse events. To minimize the potential influence of clinical status and recent vaccinations on systemic inflammation, we excluded outcome data from patients with active malignancy, as well as those from samples in patients with evident infections or vaccinations occurring within one week prior to blood collection.

### Statistical Analysis

Values are reported as mean ± SD, median (IQR), or count (%) as appropriate. Patients were divided into tertiles (small, moderate, or large infarct size) based on their highest measured CK (CKmax) level during hospitalization. Tests for linear trends in clinical characteristics across infarct size strata were performed using generalized linear regression and Jonckheere–Terpstra tests for continuous variables, and the Cochran–Armitage test for categorical variables.

For all biomarkers, mixed models were used to assess temporal changes across infarct size strata, including the interaction between infarct size and timepoint as fixed effects and patient as a random effect. Although previous studies have shown that PCSK9 inhibitors exert a neutral effect on systemic inflammatory biomarkers, the interaction between randomization group and timepoint was included as an additional fixed factor to account for any potential differences at each timepoint [15–17]. Kenward–Roger degrees of freedom were used for all calculations. No log-transformation of CRP or IL-6 concentrations was applied. The overall timepoint-by-infarct size interaction and overall randomization group-by-timepoint interaction was evaluated using Type III F-tests. Model-based estimated marginal means were used to explore pairwise comparisons between infarct size groups at each timepoint. Between-group comparisons at each timepoint were adjusted for multiple comparisons using Tukey’s method. Likewise, estimated marginal means were used to assess temporal changes within each infarct size group, providing a detailed description of biomarker trajectories. To account for multiple testing across biomarkers in the Olink panel, false-discovery-rate correction (Benjamini–Hochberg) was applied to all outcomes within each reported analysis (overall interaction, cross-sectional timepoints, and within-group changes), analyzed separately by matrix. Differentially expressed proteins were annotated to biological processes using the Human Protein Atlas (version 24.0).

Additionally, Spearman’s rank correlation coefficient was calculated for correlations between CKmax and CRP and IL-6 at baseline. All reported *P* values are two-sided. For CRP analysis, IL-6 analysis, and Spearman’s rank correlations, a *P* value < 0.05 was considered statistically significant. For Olink parameters, a *P*_adj_ < 0.05 was regarded as statistically significant. Statistical analyses were performed using R-studio version 4.4.2 (R Foundation for Statistical Computing, Vienna, Austria).

## Results

### Patient Characteristics

In total, 73 patients presenting with ACS at the Radboud university medical center underwent complete biomarker assessment (Supplementary Fig. 1). Three patients were excluded from the analysis due to, the absence of myocardial necrosis (unstable angina), a malignancy, and a vaccination within one week of follow-up assessment. Therefore, a total of 70 patients with MI were eventually included in the analysis and grouped according to infarct size. All study visits were carried out between November 2020 and November 2023. The baseline characteristics of all patients are summarized in Table 1. The median time from chest pain onset to clinical presentation was 3.6 h (1.5 to 5.9), and the median time from symptom onset to blood sampling for inflammatory biomarker assessment was 1.3 days (0.6 to 3.0). Overall, mean age was 64.8 ± 9.0 years, 56 (80.0%) were male, and 14 (20.0%) were female. Median CK levels for the small, moderate, and large infarct size groups were 106.0 U/L (74.0 to 158.0), 445.0 U/L (285.5 to 519.5), and 1884.0 U/L (1315.0 to 2265.0), respectively (*P*_trend_ < 0.001). The proportion of patients presenting with STEMI increased with a larger infarct size (*P*_trend_ < 0.001). Except for a slight imbalance in aspirin use at baseline, baseline characteristics and discharge medications (Supplementary Table 1) were evenly distributed across infarct size strata.

**Table 1:** Baseline characteristics

	Small MI (*n* = 24)	Moderate MI (*n* = 23)	Large MI (*n* = 23)	*P*_trend_	Overall (*n* = 70)
Demographics					
Age – years	66.1 ± 11.1	64.2 ± 7.5	64.1 ± 8.2	0.455	64.8 ± 9.0
Sex, female	7 (29.2%)	4 (17.4%)	3 (13.0%)	0.166	14 (20.0%)
Cardiovascular risk factors					
BMI – kg/m^2^	27.9 ± 3.8	26.3 ± 3.3	27.6 ± 3.0	0.806	27.3 ± 3.4
Hypertension	10 (41.7%)	8 (34.8%)	7 (30.4%)	0.421	25 (35.7%)
Dyslipidemia	13 (54.2%)	5 (21.7%)	8 (34.8%)	0.162	26 (37.1%)
Family history of premature CAD	12 (50.0%)	10 (43.5%)	12 (52.2%)	0.887	34 (48.6%)
Current smoking	8 (33.3%)	6 (26.1%)	6 (26.1%)	0.580	20 (28.6%)
Diabetes mellitus	2 (8.3%)	4 (17.4%)	1 (4.3%)	0.662	7 (10.0%)
Medical history					
Stroke or transient ischemic attack	4 (16.7%)	0 (0.0%)	1 (4.3%)	0.097	5 (7.1%)
Peripheral artery disease	0 (0.0%)	1 (4.3%)	0 (0.0%)	0.986	1 (1.4%)
Prior MI	3 (12.5%)	3 (13.0%)	3 (13.0%)	0.955	9 (12.9%)
Prior percutaneous coronary intervention	5 (20.8%)	3 (13.0%)	3 (13.0%)	0.460	11 (15.7%)
Medication at admission					
Aspirin	8 (33.3%)	4 (17.4%)	2 (8.7%)	0.034	14 (20.0%)
ADPRI (ticagrelor/clopidogrel/prasugrel)	3 (12.5%)	1 (4.3%)	1 (4.3%)	0.275	5 (7.1%)
Any statins	8 (33.3%)	5 (21.7%)	3 (13.0%)	0.097	16 (22.9%)
ACE inhibitor	2 (8.3%)	3 (13.0%)	2 (8.7%)	0.961	7 (10.0%)
ARB	0 (0.0%)	1 (4.3%)	1 (4.3%)	0.368	2 (2.9%)
Beta-blocker	7 (29.2%)	2 (8.7%)	4 (17.4%)	0.291	13 (18.6%)
MI characteristics					
Infarct type					
STEMI	0 (0.0%)	7 (30.4%)	17 (73.9%)	< 0.001	24 (34.3%)
NSTEMI	24 (100.0%)	16 (69.6%)	6 (26.1%)	< 0.001	46 (65.7%)
Maximum creatine kinase level – U/L*	106.0 (74.0–158.0)	445.0 (285.5–519.5)	1884.0 (1315.0–2265.0)	< 0.001	445.0 (161.0–1314.0)

Values are shown as mean ± SD, median (IQR) or count (%)

^*^ Creatine kinase levels were missing for one patient, who was stratified as having a small infarction because of a maximum measured troponin T level of 104 ng/L (Upper limit of normal: 13 ng/L)

Abbreviations: *ACE* angiotensin converting enzyme, *ADPRI* adenosine diphosphate receptor inhibitor, *ARB* angiotensin receptor blocker, *BMI* body mass index (calculated as weight in kilograms divided by height in meters squared), *CAD* coronary artery disease, *MI* myocardial infarction, *NSTEMI* non-ST-segment elevation myocardial infarction, *STEMI* ST-segment elevation myocardial infarction of apoptosis

### CRP and IL-6 Concentrations Over Time

**Fig. 1:**
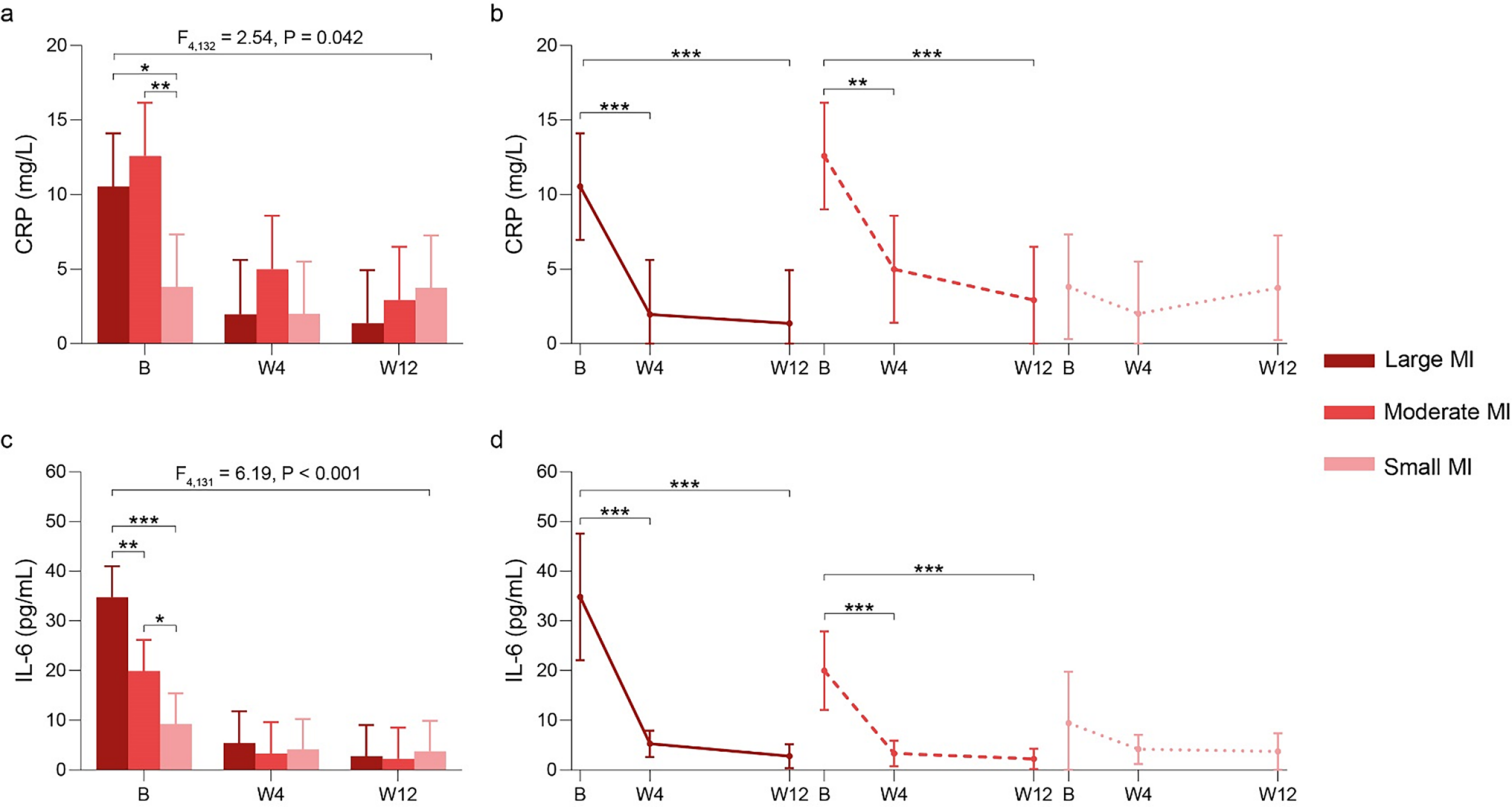
Plasma concentrations of CRP and IL-6 following myocardial infarction. Bar charts and line plots depict mean (95% CI) CRP and IL-6 concentrations. Differences at each time point **a**,**c** and changes over time **b**,**d** were derived from pairwise contrasts of the mixed model. Measurements from one patient at week 4 were excluded due to vaccination within one week prior to blood sampling (large MI stratum), resulting in *n* = 69 at week 4. Significance is indicated as follows: * *P* < 0.05; **, *P* < 0.01; *** *P* < 0.001. Abbreviations: *CRP* C-reactive protein, *IL-6* interleukin 6, *MI* myocardial infarction

Regarding CRP, a significant time-by-infarct size interaction was observed (F_4,132_ = 2.54, *P* = 0.042), indicating a significant differential temporal pattern across infarct size strata (Fig. 1a). Differences in CRP were observed only at baseline, with higher concentrations in patients with moderate and large MI compared to patients with small MI (Fig. 1a). Over time, CRP decreased significantly in patients with moderate and large MI (Fig. 1b).

Similarly, for IL-6, temporal changes differed significantly between infarct size groups (F₄_,_₁₃₁ = 6.19, *P* < 0.001). Model-based pairwise comparisons revealed a stepwise elevation in baseline IL-6 across the infarct size strata (Fig. 1c). As observed for CRP, IL-6 levels declined over time in patients with moderate and large MI. (Fig. 1d).

No significant between-group differences in CRP or IL-6 were detected from week 4 onwards (Fig. 1a,c). At week 12, the overall mean CRP was 2.72 ± 4.70 mg/L and overall mean IL-6 was 2.90 ± 6.53 pg/mL. In patients with small MI, no significant temporal changes in CRP or IL-6 concentrations were observed (Fig. 1c,d). In addition, significant correlations between CKmax and CRP (Spearman’s ρ = 0.44, *P* < 0.001) and IL-6 (Spearman’s ρ = 0.57, *P* < 0.001) were found at baseline. Of note, no significant interaction between time and randomization group was found for CRP or IL-6 concentrations.

### Targeted Proteomics using Olink’s Inflammation Panel

Out of the total 92 proteins included in Olink’s inflammation panel, IL-1α, IL-2, IL-22 RA1, beta-nerve growth factor (beta-NGF), IL-24, IL-33, IL-5, and IL-20 were excluded from the analysis due to > 80% of the samples having values below the adequate detection limit in at least one of the analyses plates. Subsequently, the expression of 84 proteins was assessed.

**Fig. 2:**
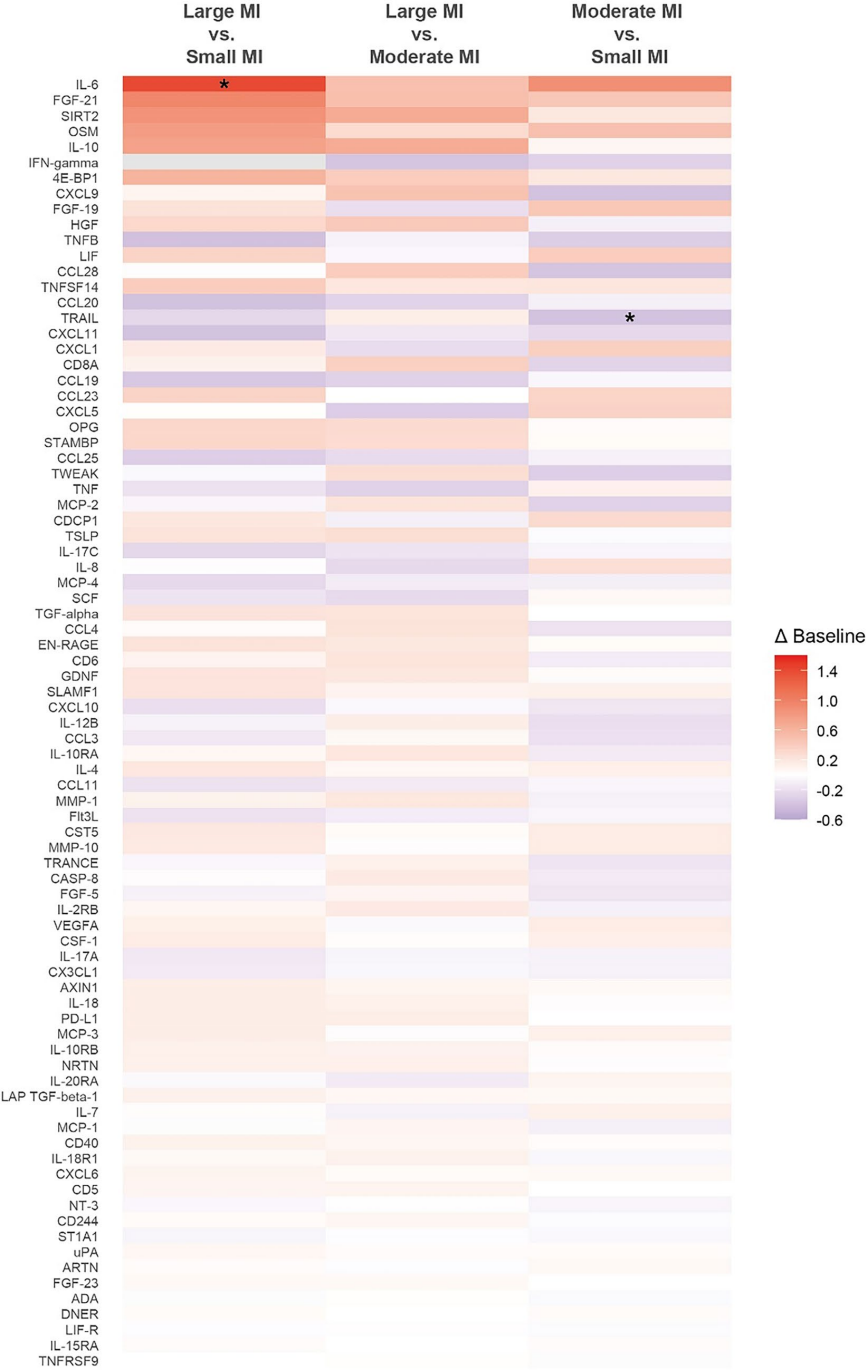
Differential protein expression during the acute phase of myocardial infarction (baseline). Heat map illustrating relative differences in baseline protein expression between infarct size strata, derived from pairwise contrasts of the mixed-effects model. Red indicates higher expression and blue lower expression. Pairwise comparisons were adjusted using Tukey’s method within each protein. To account for multiple testing, false discovery rate (FDR) correction was applied across all proteins and pairwise contrasts using the Benjamini–Hochberg procedure. An asterisk (*) indicates FDR-adjusted significance (*P*_adj_ < 0.05). Abbreviations: *MI* myocardial infarction

A significant overall interaction between timepoint and infarct size was observed for IL-6 (F_2,66_ = 10.46, *P*_adj_ = 0.009), tumor-necrosis factor-β (TNFβ, e.g. lymphotoxin alpha, F_2,66_ = 8.80, *P*_adj_ = 0.017), and TNF receptor superfamily member 9 (TNFRSF9, F_2,66_ = 7.20, *P*_adj_ = 0.041), indicating differences in protein trajectories over time across infarct size strata. Differential protein expression at baseline is illustrated in Fig. 2, showing a graded increase (indicated in red) in many inflammatory proteins with increasing infarct size. Examination of model-based comparisons at baseline demonstrated a significant higher expression of IL-6 in patients with large MI compared to patients with small MI and a lower expression of TNF-related apoptosis inducing ligand (TRAIL) in patients with moderate MI compared to patient with small MI (Fig. 2).

**Fig. 3:**
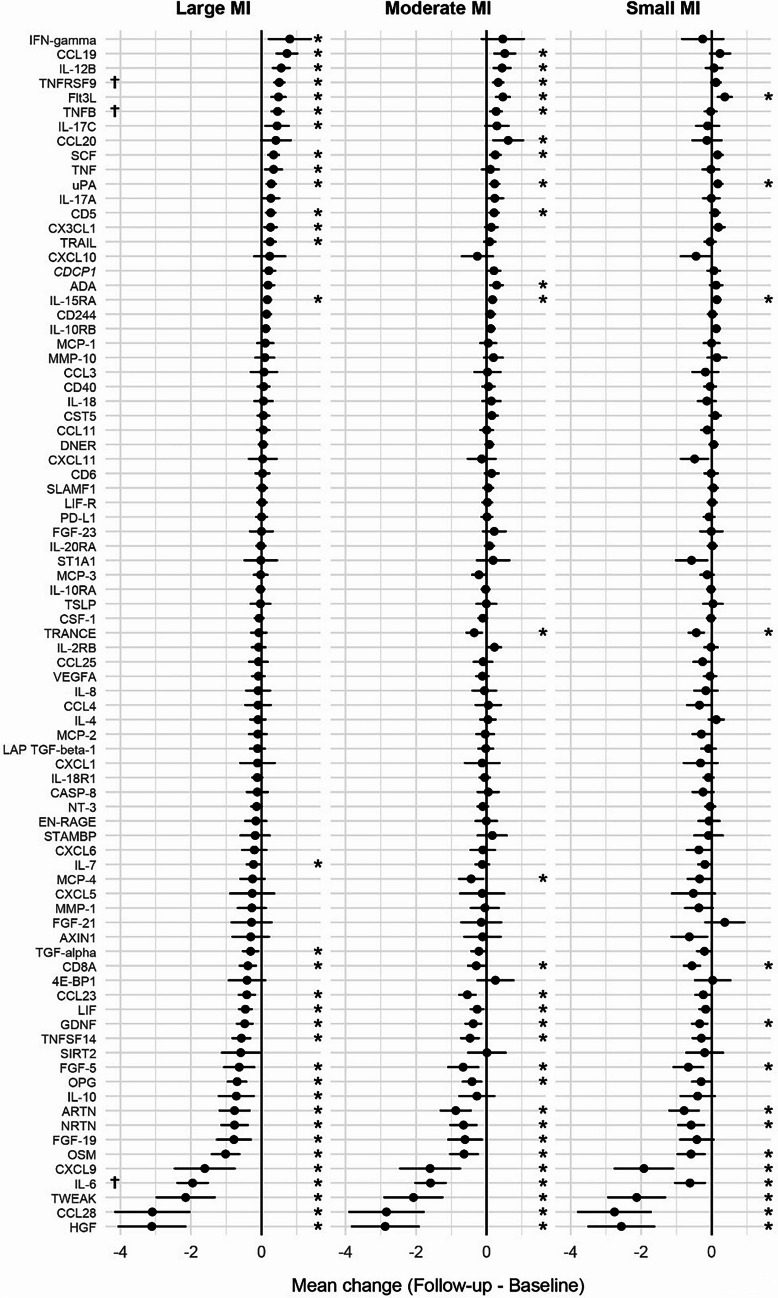
Differential change in protein expression following myocardial infarction (baseline to 12-week follow-up). Forest plots of the change in inflammatory protein expression from baseline to 12-week follow-up across infarct size strata. Negative values indicate a decrease from baseline, whereas positive values indicate an increase. To account for multiple testing, false discovery rate (FDR) correction was applied separately within each infarct size stratum using the Benjamini–Hochberg procedure. An asterisk (*) indicates FDR-adjusted significance (*P*_adj_ < 0.05). Proteins with a significant overall time-by-infarct size interaction are indicated by: ✝. Abbreviations: *MI* myocardial infarction.

Overall, many biomarkers demonstrated a significant change over time, as illustrated by infarct size strata in Fig. 3. Most significant differences over time were observed in patients with large myocardial injury. At the acute moment, main upregulated proteins included hepatocyte growth factor (HGF), CC motif chemokine ligand 28 (CCL28), tumor necrosis factor-related weak inducer of apoptosis (TWEAK), IL-6, and CXC motif chemokine ligand 9 (CXCL9). Among the main downregulated proteins were interferon gamma (IFN gamma), CCL19, IL-12B, TNFRSF9, Fms-related tyrosine kinase 3 ligand (Flt3L), TNFβ, and urokinase (uPA). Given the significant timepoint-by-infarct size interaction for IL-6, TNFβ, and TNFRSF9, Fig. 3 illustrates the direction and magnitude of within-group changes, with changes appearing progressively more pronounced across increasing infarct size.

At follow-up, no significant differential protein expression was observed among infarct size strata after multiplicity correction. Similarly, no significant randomization group-by-timepoint interaction was found for any protein.

## Discussion

We aimed to provide a detailed characterization of the systemic inflammatory response following MI across patients with varying infarct size. Our study demonstrates that patients with differing severity of myocardial injury exhibit distinct circulating inflammatory biomarker profiles during the first 12 weeks after MI, particularly involving IL-6. Even after adjustment for multiple testing across numerous biomarkers, IL-6 remained significantly upregulated in patients with large MI compared to patients with small MI. In patients with small MI, no significant temporal changes in CRP or IL-6 concentrations were found. Of interest, all patients exhibited reduced expression of uPA and upregulation of angiogenesis-related proteins (HGF and TWEAK). No significant effect of PCSK9 inhibition was detected, which aligns with previous studies investigating the effect of PCSK9 inhibitors on systemic inflammatory biomarkers [15–17].

### Circulating CRP and IL-6 Concentrations Following MI

The past few decades, CRP has emerged as parameter of adverse cardiovascular outcome, also in the acute phase of MI [1, 5, 18]. The upstream IL-1—IL-6 pathway seems the pivotal inducer of hepatic CRP production and has attracted considerable attention [1, 18]. Recent genetic studies showed that IL-6 signaling is causally related to atherosclerotic cardiovascular disease [19]. Many leukocytes, including lymphocytes, macrophage, and monocytes, as well as vascular smooth muscle cells and fibroblasts have the capability to produce IL-6, mainly upon activation by IL-1β or TNFα [7, 20, 21]. In turn, a wide range of immune- and non-immune cells are affected by IL-6. It is therefore regarded to exhibit hormone-like properties [20, 21]. The course of CRP and IL-6 following MI has been studied before. Peak concentrations of IL-6 and CRP are reached after approximately 1 and 2 days, respectively, with CRP declining more gradually thereafter [22, 23]. While serial cytokine measurements were not obtained during the first days after MI, our findings align with previous reports indicating that IL-6 peaks earlier than CRP [22, 23]. From week 4 onwards, no differences in CRP or IL-6 were observed between the infarct size groups. Interestingly, overall mean CRP was 2.72 ± 4.70 mg/L at week 12, which is higher than the widely accepted threshold of ≥ 2 mg/L to define residual inflammatory risk [24]. These findings are consistent with *Carrero *et al., who measured high-sensitivity CRP (hsCRP) several weeks post-MI [25]. Additionally, post-MI hsCRP remained prognostic: concentrations ≥ 2 mg/L were linked to an elevated risk of major adverse cardiovascular events over a median 3.2-year follow-up [25]. Of interest, patients with small infarct size did not show a significant elevation in CRP, suggesting that even early CRP measurements may be representative of residual inflammatory risk.

### Infarct Size and Inflammatory Markers

We found a clear correlation between infarct size and CRP dynamics, and notably, IL-6. These findings suggest that the magnitude of myocardial necrosis is an important determinant in the elevation of inflammatory biomarkers. This assumption is consistent with findings of *Ritschel *et al., who reported significantly higher levels of CRP and IL-6 in patients within the upper quartiles of peak troponin T. They also observed significant inverse correlations between left-ventricular ejection fraction (LVEF) after STEMI and plasma levels of IL-6 and CRP. The prospective MARINASTEMI trial assessed the relationship between IL-6 levels following STEMI and cardiac magnetic resonance-derived infarction metrics [26]. Higher IL-6 concentrations were significantly associated with larger infarct sizes, greater microvascular obstruction and lower LVEF. Regarding hsCRP, only microvascular obstruction was correlated [26]. Moreover, results from the SOLID-TIMI 52 trial showed that increased IL-6 after ACS is significantly associated with the risk of serious adverse cardiovascular events, even after controlling for hsCRP [6]. Taken together, acute IL-6 measurements might provide greater additional prognostic insights compared to CRP.

### Inflammatory Protein Expression Following MI

The Olink proteomics platform allows for simultaneous measurement of a wide range of inflammatory proteins. After controlling for multiplicity, baseline IL-6 expression increased significantly with the extent of myocardial injury. These observations emphasize IL-6 as a potential target for anti-inflammatory therapy in patients with large MI and corroborate our findings regarding plasma concentrations. In addition, a wide range of proteins demonstrated change over time. Comprehensive, serial, multi-biomarker profiling in the immediate post-ACS window remains scarce. In a previous study, higher levels of IL-6 were observed in patients with ACS compared to those with chronic coronary syndrome (CCS) prior to cardiac surgery [27]. In the same study, additional analyses revealed no significant differences in cytokine levels between ACS and CCS patients after three months, suggesting that most cytokines return to stable levels following ACS [27]. *Björkenheim *et al. applied the Olink inflammation panel to a small cohort of STEMI patients at baseline and after four to six weeks of follow-up [28]. Correspondingly to our study, CCL19 was downregulated, while OSM, HGF, and IL-6 were among the upregulated proteins [28]. We observed markedly more differentially expressed proteins, even in patients with small MI. Our patient groups were considerably larger, but the findings might also suggest that it takes more than six weeks for these proteins to normalize.

Aside from IL-6, key biological processes linked to the upregulated differentially expressed proteins during MI include apoptosis (TWEAK), regulation of cell growth and IL-6 production (OSM), and chemotaxis of T-cells (CCL28 and CXCL9). As opposed to TWEAK, HGF exerts anti-apoptotic effects and is therefore considered cardioprotective [29–31]. Both proteins promote angiogenesis and constitute to tissue regeneration [29, 30, 32]. Interestingly, TWEAK and HGF were also upregulated in patients with small MI, suggesting that even limited ischemic injury is sufficient to trigger the release of pro-angiogenic signals. This contrasts with the absence of an increase in CRP or IL-6 concentrations in patients with small MI. While CRP is predominantly produced in the liver in response to systemic IL-6 signaling, TWEAK and HGF are produced by a variety of cell types and may be released locally in response to tissue injury and inflammation.

Principal biological pathways connected to the downregulated genes during MI involve proliferation of early hematopoietic cells (Flt3L), activating effector immune cells (IFN gamma), chemotaxis for T-cells and B-cells (CCL19), cytotoxicity (TNFβ and TNFRSF9), stimulating growth of activated T cells and natural killer cells (IL-12B), and fibrinolysis (uPA). TNFβ and TNFRSF9 exhibited a significant temporal interaction with infarct size, with more pronounced increases over time in patients with larger infarcts, which may reflect activation of cytotoxic pathways in the context of greater myocardial injury.

The reduced uPA expression likely contributes to the increased coagulable state observed following MI [33]. In addition to fibrinolysis, uPA also plays an important role in vascular remodeling and wound healing [34]. Unfortunately, the cell membrane-associated receptor for uPA (uPAR) is not included in the analysis. uPAR is expressed on a variety of cells, including fibroblasts, macrophages, monocytes, and endothelial cells [34]. On the cell surface, uPA binds uPAR with high affinity, and the uPA/uPAR system plays a pivotal role in tightly localized extracellular matrix degradation and plasmin-mediated pericellular proteolysis [35, 36]. In addition, the uPA/uPAR system contributes to localized cell migration, differentiation, and proliferation [35, 36]. Under physiological conditions, uPAR expression is limited, while injury and inflammation lead to marked upregulation [36]. In MI, uPAR expression has been shown to be upregulated on circulating monocytes, which was associated to enhanced cell adhesion to the endothelium [37]. It can therefore be hypothesized that the inflammatory response promotes increased binding and sequestration of uPA by uPAR-expressing leukocytes, potentially reducing extracellular uPA availability for fibrinolysis. This proposed mechanism warrants further investigation.

### Targeting Inflammation

Several studies have investigated strategies aimed at modulating the initial inflammatory surge that occurs following MI by targeting the IL-1 and IL-6 pathway. The VCU-ART trials assessed the use of Anakinra, an IL-1 receptor antagonist, in the wake of STEMI [38–40]. In the two pilot studies, Anakinra administration was associated with amelioration of left ventricular remodeling and a blunted increase in hsCRP [38, 39]. In the main trial, Anakinra-treated patients experienced significantly fewer heart failure–related events, despite no differences in LVEF [40]. An analogue protocol was used in the MRC-ILA study, which enrolled NSTEMI patients within 48 h of symptom onset. Interestingly, inflammatory biomarkers were suppressed during treatment, but hsCRP was elevated compared to placebo at 30 days [41]. Though not powered for clinical endpoints, reported major adverse cardiovascular events were higher in Anakinra-treated patients after one year. Differences in outcome may be attributable to delayed initiation of IL-1 inhibition, a relatively brief 14-day course of treatment, and the exclusive enrollment of NSTEMI patients, who may present with a lower inflammatory burden. Regarding IL-6 receptor antagonism, the ASSAIL-MI trial demonstrated that a single dose of tocilizumab administered within hours after symptom onset, reduced hsCRP levels, microvascular obstruction, and improved the myocardial salvage index in STEMI patients [8].

Evidence from the VCU-ART and ASSAIL-MI trials indicates that timely initiation of anti-inflammatory therapy may be beneficial [8, 38–40]. However, the MRC-ILA trial suggests that the therapeutic advantage may not extend to all patients [41]. Careful selection of patients with large inflammatory burden and immediate initiation of anti-inflammatory therapy seems of importance [1]. Our study highlights the prompt rise in IL-6, which reaches higher levels with increasing infarct size. This supports the IL-6 pathway as biomarker to identify patients with the highest inflammatory response, and as potential target for expeditious anti-inflammatory therapy in patients with high IL-6 values. However, anti-inflammatory therapy may also interfere with the inflammatory response required for tissue repair. Therefore, further studies are needed to determine whether this approach has adverse effects on myocardial healing in certain patients. Ultimately, large clinical trials are warranted to assess its impact on clinical outcomes. The ongoing ARTEMIS trial (NCT06118281), which aims to enroll 10.000 patients, is evaluating whether early initiation of ziltivekimab, a monoclonal antibody targeting IL-6, reduces major adverse cardiovascular events after MI. Study completion is anticipated in September 2026.

### Limitations

A few limitations should be considered. Although our sample size exceeded that of previous studies assessing temporal changes after MI, the total number of patients remained relatively small and may limit the power to detect between-group differences [28]. Larger cohorts would be valuable to validate our findings and further characterize group-specific patterns. Also, patients were included as soon as possible during the index hospitalization. However, enrollment was not restricted to a specific timeframe from symptom onset. As a result, peak values may have been missed in some patients. In addition, patients were stratified into tertiles based on CKmax. The use of laboratory cut-off values to assess infarct size and subsequent inflammatory burden will require prospective validation. Furthermore, no cardiac imaging was performed to confirm the extent of myocardial necrosis. Nonetheless, peak CK levels have been demonstrated to correlate strongly with final infarct size [12–14].

## Conclusion

Among patients with MI, the extent of myocardial necrosis is associated with a larger inflammatory burden. Comprehensive biomarker assessment identified IL-6 as being significantly over-expressed in patients with larger myocardial injury. Significant downregulation of uPA and upregulation of angiogenesis-related proteins were observed across all infarct size strata, underscoring the biological relevance of these processes. The current findings support IL-6 as a therapeutic target for anti-inflammatory intervention to potentially improve clinical outcomes and suggest that patients with larger myocardial injury may derive greater benefit.

## Supplementary Information

Below is the link to the electronic supplementary material.

Supplementary file1 (DOCX 169 KB)

## Data Availability

The data that support the findings of this study are available from the corresponding author upon reasonable request.

## Abbreviations

ACS: Acute coronary syndrome
Beta-NGF: Beta-nerve growth factor
CCS: Chronic coronary syndrome
CCL: CC motif chemokine ligand
CXCL: CXC motif chemokine ligand
CK: Creatine kinase
CKmax: Maximum measured creatine kinase level
CRP: C-reactive protein
Flt3L: Fms-related tyrosine kinase 3 ligand
hsCRP: High-sensitivity C-reactive protein
HGF: Hepatocyte growth factor
IL: Interleukin
LVEF: Left-ventricular ejection fraction
MI: Myocardial infarction
NPX: Normalized Protein eXpression
NSTEMI: Non-ST-elevation myocardial infarction
OSM: Oncostatin M
PCSK9: Proprotein convertase subtilisin/kexin 9
STEMI: ST-elevation myocardial infarction
TNF: Tumor necrosis factor
TNFRSF: TNF receptor superfamily member
TRAIL: TNF-related apoptosis-inducing ligand
TWEAK: Tumor necrosis factor-related weak inducer of apoptosis
uPA: Urokinase
uPAR: Urokinase receptor
