# Supplementary material for: The Immediate Impact of Infarct Size on the Systemic Inflammatory Response: IL-6 as Central Mediator Identified through Biomarker and Proteomic Profiling
Source: J Cardiovasc Transl Res. 2026 Jun 29;19(1):79. doi: 10.1007/s12265-026-10801-8 (PMC13314833; doi:10.1007/s12265-026-10801-8)

## **Supplementary table 1.** Medication at discharge

| **Medication** | **Small MI**  **(n = 24)** | **Moderate MI**  **(n = 23 )** | **Large MI**  **(n = 23)** | **P_trend_** | **Overall**  **(n = 70)** |
| --- | --- | --- | --- | --- | --- |
| Aspirin | 23 (95.8%) | 23 (100.0%) | 23 (100.0%) | 0.226 | 69 (98.6%) |
| ADPRI (ticagrelor/clopidogrel/prasugrel) | 24 (100.0%) | 23 (100.0%) | 23 (100.0%) | NA | 70 (100.0%) |
| Statins | 24 (100.0%) | 23 (100.0%) | 23 (100.0%) | NA | 70 (100.0%) |
| PCSK9 inhibitor (evolocumab) | 11 (45.8%) | 11 (47.8%) | 11 (47.8%) | 0.890 | 33 (47.1%) |
| ACE inhibitor | 21 (87.5%) | 17 (73.9%) | 21 (91.3%) | 0.735 | 59 (84.3%) |
| ARB | 0 (0.0%) | 1 (4.3%) | 1 (4.3%) | 0.368 | 2 (2.9%) |
| Beta-blocker | 23 (95.8%) | 20 (87.0% | 23 (100.0%) | 0.553 | 66 (94.3%) |

Values are shown as count (%)

Abbreviations: ACE, angiotensin converting enzyme; ADPRI, adenosine diphosphate receptor inhibitor; ARB, angiotensin receptor blocker; MI, myocardial infarction; NA, not applicable; PCSK9, proprotein convertase subtilisin/kexin type 9

## **Supplementary figure 1.** Flow of patients


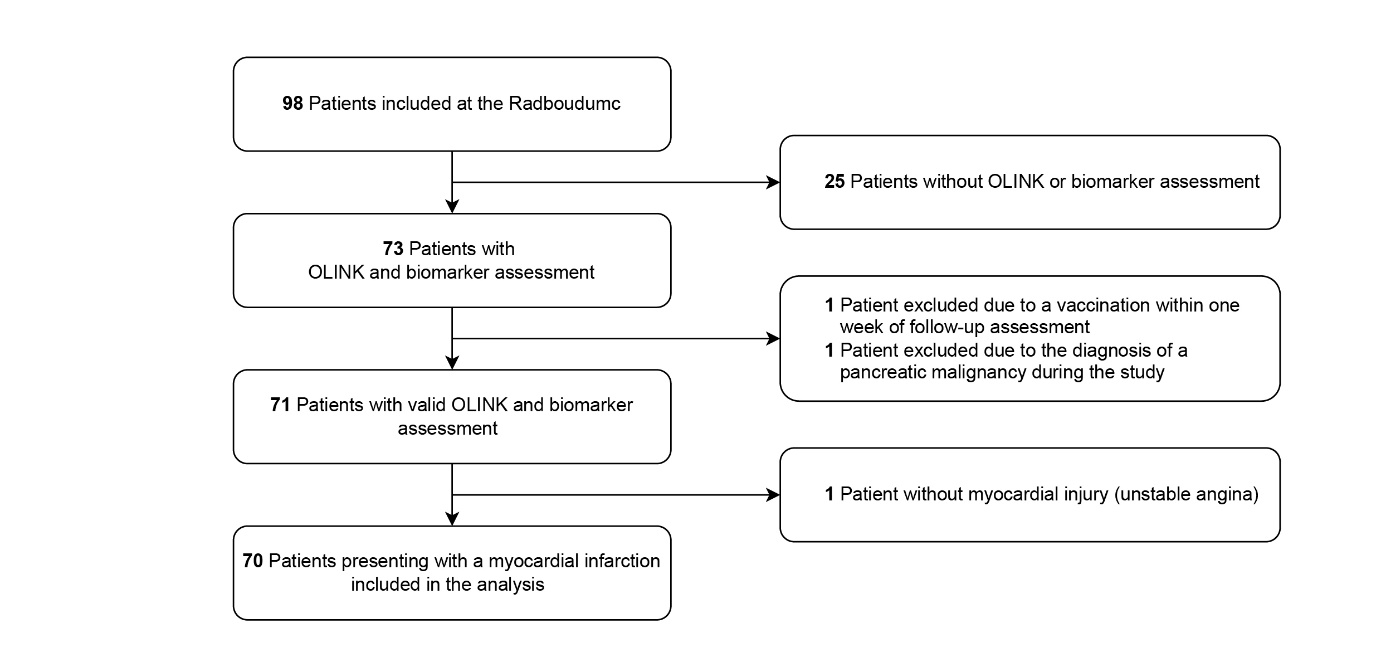

Supplement: Supplementary file 1 — Supplementary file1 (DOCX 169 KB) [file 12265_2026_10801_MOESM1_ESM.docx]
